# Profiling Dating Apps Users: Sociodemographic and Personality Characteristics

**DOI:** 10.3390/ijerph17103653

**Published:** 2020-05-22

**Authors:** Ángel Castro, Juan Ramón Barrada, Pedro J. Ramos-Villagrasa, Elena Fernández-del-Río

**Affiliations:** 1 Facultad de Ciencias Sociales y Humanas. Universidad de Zaragoza. Calle Atarazanas, 4. 44003 Teruel, Spain; castroa@unizar.es; 2 Facultad de Ciencias Sociales y del Trabajo. Universidad de Zaragoza. Calle Violante de Hungría, 23. 50009 Zaragoza, Spain; pjramos@unizar.es (P.J.R.-V.); elenario@unizar.es (E.F.-d.-R.)

**Keywords:** dating apps, Tinder, Grindr, Big Five, Dark Core, university students

## Abstract

The development of new technologies, the expansion of the Internet, and the emergence of dating apps (e.g., Tinder, Grindr) in recent years have changed the way to meet and approach potential romantic and/or sexual partners. The recent phenomenon has led to some gaps in the literature on individual differences (sociodemographic variables and personality traits) between users (previous and current users) and non-users of dating apps. Thus, the aim of this study was to analyze the relationship between using dating apps, sociodemographics (gender, age, sexual orientation, and relationship status), and bright and dark personality traits. Participants were 1705 university students (70% women, 30% men), aged between 18 and 26 (*M* = 20.60, *SD* = 2.09), who completed several online questionnaires. Through multinomial logistic regression analyses, it was found that men, older youth, and members of sexual minorities were more likely to be current and previous dating apps users. Being single and higher scores in open-mindedness were associated with higher probability to be current dating apps user. The dark personality showed no predictive ability. The discussion highlights the usefulness of knowing and considering the sociodemographic background and the characteristics of personality patterns in the design and implementation of preventive and promotion programs of healthy romantic and sexual relationships to improve people’s better health and well-being.

## 1. Introduction

The development of new technologies has changed people’s lives, affecting both their intimacy and how they relate to others. Over the past two decades, the successive popularization of the Internet and smartphone use has changed the way potential couples approach each other for millions of people worldwide. More recently, the use of location-based real-time dating apps has been extended (e.g., Tinder, Grindr), designed to maximize social, romantic, and sexual connections between strangers who are geographically nearby [1,2,3,4].

The emergence and development of dating apps have attracted considerable research interest over the past five years. Usage patterns and user profiles have both been studied, as well as the advantages and disadvantages they may have for the mental and relational health of those who use them [5,6]. Among the advantages, their portability, availability, locatability, and multimediality [2] have been highlighted, aspects that facilitate the immediate interaction with possible partners. However, there have also been risks associated with the use of apps, which can affect both mental (e.g., problematic use, related to dark personality patterns) [7] and relational health (e.g., infidelity, performance of risky behaviors, sexual victimization) [5,8].

Previous literature has confirmed that the use of dating apps is associated with different sociodemographic and personality factors. Concerning sex, it has traditionally been considered that men used dating apps more [9]. Currently, it is considered that, although men use them more and more intensely, women use them more selectively and effectively, achieving a greater number of encounters with other users [3,4]. Regarding age, previous studies have mostly evaluated the use of dating apps in college youth aged 18 to 24 [3]. For instance, Shapiro et al. [10] found that 40% of undergraduate students, aged 18–26, used Tinder. However, various investigations suggest that the average age of users could be somewhat older, even up to 31 [1,2,9]. Thus, we expected to find a direct relationship between the age of college students and the use of dating apps.

Sexual orientation also influences the use of these applications. Several studies have found greater use in people of sexual minorities than among heterosexuals [11,12]. Further, it has been emphasized that being able to contact and communicate online can be particularly useful for people of these minorities who have trouble expressing their sexuality and/or finding a partner [13]. Finally, and although there is a stereotype that dating apps are used by singles, several studies have found that a remarkable percentage of users, between 18% and 25%, had a stable partner [4,14]. Nevertheless, it seems that these people use dating apps for different purposes than singles’ reasons (e.g., infidelity) [15].

Personality traits play a key role in understanding sexuality and have been essential in the design of sexual health preventive and promotion programs [16]. In the psychosocial area, the personality model that has shown a stronger relationship to a variety of important life outcomes is the five-factor model or the Big Five [17], a taxonomy of five personality traits whose labels can differ according to the authors’ denomination (Neuroticism or Negative Emotionality, Extraversion, Openness to Experience or Open-Mindedness, Agreeableness, and Conscientiousness). Thus, for example, the relationship between the Big Five and certain areas of sexuality such as the performance of risky behaviors, sexual functioning, or sexual assault has been studied [16]. However, there is hardly any research to analyze the phenomenon of dating apps in relation to the Big Five traits. The only reference is the study of Timmermans and De Caluwé [18], who found that young single dating apps users scored higher in Extraversion and Open-Mindedness than non-users and obtained lower scores in Conscientiousness.

In recent years, in parallel with the traditional Big Five paradigm, other proposals have emerged focused on the malevolent side of personality, which may be of special interest for the understanding of sexual phenomena [7]. Although some authors defend a multidimensional approach of these socially aversive personality traits (narcissism, Machiavellianism, psychopathy, and sadism), recent evidence about a single common factor, the so-called “Dark Core” [19], based on theoretical and methodological reasons, has gained momentum [19,20,21,22,23]. All of these traits share callousness [24] and the tendency to interpersonal exploitativeness [25]. Previous research found that this dark side of personality was associated with poor quality relationships [26]. Regarding mating behavior, it has been found that people with higher scores in dark personality traits had a less restrictive sociosexuality, more sexual partners, and a greater orientation to short-term mating and casual sexual relationships [27,28]. Concerning the use of dating apps and dark personality, the main conclusions of the few available studies can be summarized in [7,15]: (1) the role of the Dark Core as a single dimension has not been evaluated, but instead, the relationships with the different dark personality traits have been explored; (2) the associations are mediated by the reasons for using the applications, which leads to different relationships depending on the different nature of the personality traits; (3) despite having found partial associations with some patterns (i.e., Machiavellianism), the role of psychopathy has been highlighted, finding higher scores in this personality trait among dating app users than among non-users.

Perhaps due to the recent expansion of the dating apps phenomenon, the existing literature has some gaps. When analyzing the uses and users of dating apps, people who used them at some point and no longer use them have not been included in the same study, nor have they been compared to current users (at the time of study or for a previous short period) [4,8,9,15,18]. To detect possible differences between the two user profiles and to determine their correlates, it would be interesting if research consider both perspectives. In the case of the relationship between the use of dating apps and personality traits, some partial studies either analyze the relationship taking into account only the traits of the Big Five [18] or only the traits of the Dark Tetrad [7]. Only one study has simultaneously contemplated both sides of personality [15]. However, this study is aimed at determining the use of dating apps based on the relational status of the participants and the patterns of infidelity and not so much to explore the differences in individual tendencies between users and non-users.

To fill these gaps, the objective of this study was to analyze the relationship between the previous use and current use (the last three months) of dating apps and the personality traits (Big Five and Dark Core) in a sample of young college students. In this way, we aim to examine the relationships between the use of these applications and personality traits, as well as to know which of those traits can predict the use of dating apps. Further, the predictive role of different sociodemographic variables, such as gender, age, sexual orientation, and relational status, is explored. Knowing the individual tendencies of users of this type of apps can be useful for the design and implementation of preventive and promotion programs for mental health and healthy relationships, both romantic and sexual, in this group.

## 2. Materials and Methods

### 2.1. Participants and Procedure

The initial sample comprised 1996 participants. Four inclusion criteria were used: (1) studying a university degree at the time of data collection (76 participants excluded); (2) aged between 18 to 26 years, according to criteria from previous studies with university samples [29,30,31] (128 participants excluded); (3) labeling themselves as woman or man (13 participants excluded; the small sample size of this group prevented us from incorporating these participants to our analyses); and (4) correctly answering a control question (74 participants excluded; see below).

Considering all these criteria, the final sample included 1705 university students (70% women, 30% men), aged between 18 and 26 (*M* = 20.60, *SD* = 2.09). Of the participants, 70.1% described themselves as heterosexual, 22.5% as bisexual, 5.8% as homosexual, and 1.6% as other orientations. Due to the small sample sizes of non-heterosexual groups, those participants were combined into a sexual minority category (29.9%). Concerning relationship status, 52.9% of the participants had a partner, with an average relationship duration of 26.1 months (*SD* = 22.6), and 47.1% had no partner.

Regarding the procedure, data were collected in December 2019 using a Google Forms survey. To reach participants, a link to the survey was distributed through the e-mail distribution lists of the students of the authors’ university. Participants provided informed consent after reading the description of the study, where the anonymity of the responses was clearly stated. The survey remained open for 14 days. This procedure was approved by the Ethics Review Board for Clinical Research of the region (PI18/058).

### 2.2. Measures

#### 2.2.1. Sociodemographic and Dating App Use Questionnaire

We asked participants about their gender (woman, men, other), age, sexual orientation (heterosexual, homosexual, bisexual, other), and whether they were in a relationship (if they were, for how long). We also asked them whether they had ever used any dating app (Tinder, Grindr or similar) and whether they had used any in the past three months before the study. People who answered “No” to both questions were identified as “nonusers”, those who only answered “Yes” to the second one were labeled as “current user”, and the rest of the sample were identified as “previous users”.

#### 2.2.2. Short Form of the Big Five Inventory–2

This instrument [32] (the short form the original BFI-2) [33] has 30 items that assess the Big Five domains: Negative Emotionality (e.g., “[I am someone who...] is moody, has up and down mood swings”; α = 0.75—all reported alpha values correspond to those observed in the current sample); Extraversion (e.g., “is outgoing, sociable”; α = 0.71); Open-Mindedness (e.g., “is curious about many different things”; α = 0.73); Agreeableness (e.g., “is compassionate, has a soft heart”; α = 0.68); and Conscientiousness (e.g., “is systematic, likes to keep things in order”; α = 0.75). These items are rated on a five-point scale, ranging from 1 = *disagree strongly* to 5 = *agree strongly*. The Spanish translation was provided by the first author of the original version of the BFI-2.

#### 2.2.3. Dark Factor of Personality–16

This instrument [20] (a short form of the full 70-item version) has 16 items that assess the dark factor of personality with a single component (e.g., “People who mess with me always regret it”; α = 0.75). These items are rated on a five-point scale, ranging from 1 = *strongly disagree* to 5 = *strongly agree*. Following the original instructions, items were presented in random order for each participant [20]. The translation into Spanish was performed for the present research. In our case, three of the co-authors, all native Spanish speakers, translated the scale from English to Spanish, reviewed the translation together, and agreed on a single version of the scale. Finally, a native professional translator reviewed the correspondence between the English and Spanish versions, which agreed with the translated version. The Spanish version can be seen in Appendix A.

#### 2.2.4. Control Question

Embedded in the questionnaire and to check whether the participants paid enough attention to the wording of the items, we introduced an item asking the participants to respond to it with *strongly disagree*. Those participants responding with an option different from the one requested could be considered distracted.

### 2.3. Data Analyses

Firstly, we computed descriptives and associations between the different variables. The correlations between dichotomous variables (gender, relationship status, and sexual orientation) with age and the six personality scores were transformed to Cohen’s *d* [34]. The effect size measure for the associations between the dating apps use groups (never, previous, current) and age and the six personality scores was the *R* statistic from the ANOVA model of means comparison. The association between the dating apps use groups and dichotomous variables was quantified with Cramer’s *V*. We chose the effect measure which we considered to allow a potentially easier interpreation of the results [35].

Secondly, we computed a multinomial logistic regression models, with the dating apps use groups as the criteria—nonusers as the reference group—and gender, relationship status, sexual orientation, age, and personality scores as the predictors. As the metric of the personality scores is not easy to interpret, we standardized them before the regression. By doing so, the odds ratio coefficients of personality variables indicate the change in the odds ratio for increments in units of one standard deviation.

The analyses were performed with R 4.0.0 (R Foundation for Statistical Computing, Vienna, Austria) [36]. No missing data were present in our database. The open database and code files for these analyses are available at the Open Science Framework repository (https://osf.io/wjuh6/).

## 3. Results

The associations among the different variables, with the descriptives, can be seen in Table 1 and Table 2. We will focus our attention on the relationship between dating apps use and sociodemographic characteristics and personality scores.

Of the participants, 71.5% (*n* = 1219) were nonusers, 15.8% (*n* = 270) were previous dating apps users, and 12.7% (*n* = 216) were current users. All sociodemographic variables were associated with the dating apps users groups. With respect to gender, for women, the distributions by group was *p*_nonuser_ = 0.75, *p*_previous_ = 0.15, and *p*_current_ = 0.10; for men, *p*_nonuser_ = 0.65, *p*_previous_ = 0.17, and *p*_current_ = 0.18; χ^2^(2) = 23.25, *p* < 0.001, *V* = 0.12. For those in a relationship, *p*_nonuser_ = 0.79, *p*_previous_ = 0.17, and *p*_current_ = 0.04; for single participants, *p*_nonuser_ = 0.65, *p*_previous_ = 0.15, and *p*_current_ = 0.20; χ^2^(2) = 100.51, *p* < 0.001, *V* = 0.24. For heterosexual participants, *p*_nonuser_ = 0.79, *p*_previous_ = 0.13, and *p*_current_ = 0.08; for sexual minority participants, *p*_nonuser_ = 0.55, *p*_previous_ = 0.23, and *p*_current_ = 0.23; χ^2^(2) = 108.64, *p* < 0.001, *V* = 0.25. Age was associated with the dating apps users groups, with previous users being the older ones (*M* = 21.70, *SD* = 2.16) and nonusers the youngers (*M* = 20.31, *SD* = 1.98), *F*(2, 1702) = 53.85, *p* < 0.001, *R* = 0.24.

Personality means differed by dating apps users group for all considered variables (all *p*s ≤ 0.017), except for Extraversion, *F*(2, 1702) = 0.00, *p* = 0.998. All effect sizes could be considered as rather small (*M*_R_ = 0.07, range [.00, 0.10]). The higher associations were with respect to Open-Mindedness (higher mean for current users) and Conscientiousness (higher mean for nonusers). We note that Conscientiousness and Dark Core showed a high negative correlation (*r* = –0.59, *p* < 0.001).

Results of the multinomial logistic regression models are shown in Table 3. The explanatory capacity of the model was moderate (Nagelkerke’s pseudo-*R*^2^ = 0.25, McFadden’s pseudo-*R*^2^ = 0.14). The explanatory ability was basically provided by the sociodemographic information. Being a member of a sexual minority greatly increased the probability of dating apps use (*OR*_previous_ = 3.08, *p* < 0.001; *OR*_current_ = 4.11, *p* < 0.001). Men had a higher probability of use (*OR*_previous_ = 1.44, *p* = 0.029; *OR*_current_ = 1.71, *p* = 0.002). Increments in age were associated with increments in the probability of use (*OR*_previous_ = 1.42, *p* < 0.001; *OR*_current_ = 1.27, *p* < 0.001). Being single showed a very interesting result, as it had an an important impact on the probability of being a current user (*OR*_current_ = 6.48, *p* < 0.001), but not with being a previous user (*OR*_previous_ = 1.22, *p* < 0.177). To better understand the relevance of these variables, we computed the probability of belonging to each group for an 18-year-old heterosexual woman in a relationship and for a 26-year-old single non-heterosexual man (both with mean scores in all personality variables). For that woman, *p*_nonuser_ = 0.95, *p*_previous_ = 0.04, and *p*_current_ = 0.01; for that man, *p*_nonuser_ = 0.12, *p*_previous_ = 0.45, and *p*_current_ = 0.44. All of the personality scores showed statistically non-significant coefficients (*OR*s in the range [0.86, 1.19], *p*s ≥ 0.058), except for Open-Mindedness with current use (*OR*_previous_ = 1.22, *p* = 0.026).

Given the relevant correlation between Agreeableness and Dark Core, which could lead to some problems of multicollinearity, we tested a model without dark personality scores. If multicollinearity was a concern, the pattern of results would be changed for this reduced model, but that was not the case. For the full model, the results for Agreeableness were as follows: *OR*_previous_ = 0.95, *p* = 0.555; *OR*_current_ = 1.00, *p* = 0.989; and for the reduced model: *OR*_previous_ = 0.96, *p* = 0.589; *OR*_current_ = 0.90, *p* = 0.215. We did not test a model without Agreeableness as we considered that, theoretically, it made no sense to exclude one of the dimensions of the Big Five.

## 4. Discussion

The emergence and popularization of dating apps in recent years have changed the way potential romantic and/or sexual partners meet and interact. Due to the recency and relevance of this phenomenon, it is necessary to deepen our knowledge about the profile of dating apps users. Although some studies have pointed out that the use of these applications varies depending on certain sociodemographic variables and personality traits, such studies are still few, and they present partial analyses, significant limitations in sampling, and their results are inconclusive. Therefore, the objective of this study was to analyze the relationship between the use of dating apps—previous and current—and the personality traits (bright and dark), also taking into account the role of sociodemographic variables such as gender, age, sexual orientation, and relational status, in a sample of young university students.

Of the participants, 71.5% were nonusers of dating apps, 15.8% were previous users, and 12.7% were current users (in the last three months). This is a prevalence of medium use, compared with that found in other studies [2,3,18], although it should be noted that, in these studies, sampling was aimed at finding people who used dating apps or it excluded those who had a partner, even with convenience samples. Therefore, although there are different reasons for the lower prevalence of dating app use in this study compared with those in previous works (e.g., participants’ age, proportion of people with partners, cultural differences), we consider that our sampling allows us to better estimate the actual percentage of users of these applications.

From the data obtained, a sociodemographic profile of dating app users can be drawn among young Spanish university students. Individuals who are members of sexual minorities, men, and older youths are more likely to use dating apps. Although the results were expected in accordance with the previous literature, it can be stated that these characteristics are consistent for both groups of users (previous and current use) and both in the bivariate and the regression analyses performed.

The past and current likelihood of using dating apps in people from sexual minorities was more than three times greater than that of heterosexual people, in line with existing evidence. As has already been shown, dating apps are a resource widely used by people from sexual minorities, especially those who have more difficulty expressing their sexuality and/or finding a partner [11,12,13].

Our data support that men use dating apps more than women, as appears in most studies collected in the review of Anzani et al. [1]. As for age, the data revealed that the older the person is, the more likely they are to have used or to use dating apps currently. In the same vein, previous studies found a higher current use in older college students, noting that the phenomenon of dating apps is more prevalent among slightly older youths [1,2,9]. Concerning previous use, it seems logical to think that older youths, due simply to their lifetime, have been more likely to have used a dating app.

Concerning relational status, the results are especially interesting. Being single greatly increased the likelihood of being a current user of dating apps, but not of being a previous user. There exists a stereotype that considers that these apps are used only for casual sex [9] and that dating app users are not interested in long-term relationships. If that was the case, previous users should still be single to a larger extent. These results indicate that dating apps can be used to find long-term relationships or that looking for casual sex is not incompatible with seeking a romantic relationship [37].

We found that 4% of current users were in a relationship. This could be due to the fact that we considered as “current” those who had used in the last three months, so those participants could be single while using the apps, but not when responding to the questionnaires. Other options are people cheating on their partners [15] or in a consensually non-monogamous relationship. Other studies have found that about 20% of dating app users are in a committed relationship [4,38]. Further research is needed to clarify that important difference.

The relationships found between personality traits and the use of dating apps allow us to draw two relevant and surprising conclusions concerning the previous literature, which we will discuss below. First, although all dimensions—but Extraversion—showed statistically significant, although small, associations with dating app use group, those effects disappeared in the regression model. This lack of effect was found for the Big Five domains and for dark personality. The only exception was Open-Mindedness, which emerged as the only personality trait associated with the current use of dating apps, in line with previous studies [18].

Some explanations are plausible to justify why the Open-Mindedness effect was not the same for previous and current use. First, it could be simply interpreted as statistical noise. Second, we cannot discard that some features of Open-Mindedness (i.e., better tolerance of change) have a higher predictive capacity when the criteria refer to more recent behaviors (i.e., use of dating apps in the last three months). To clarify the predictive value of personality traits, future research should examine in greater depth the role of the Big Five in explaining different types of dating apps use (never users vs. experimental users vs. regular users).

The Dark Core of personality was not a significant predictor of previous and current use of dating apps, contrary to previous research [7,15]. This disparity may be due to the conception of dark personality and, consequently, the way of assessing it. Preceding studies addressed the dark personality from a multidimensional approach, using scales that assess four different dark personality traits (i.e., narcissism, Machiavellianism, psychopathy, and sadism). As we mentioned, we conceived the dark personality as a single latent factor called Dark Core, according to the most recent evidence about the theoretical and empirical overlap among the dark traits [19,20,21,22,23]. Further, previous studies [7,15] used the Short Dark Triad-3 (SD-3) [39]. This instrument includes an item that, while measuring psychopathy, clearly overlaps with high sociosexual orientation (“I enjoy having sex with people I hardly know”). Therefore, higher scores for apps users may be indicative of higher dark personality or higher sociosexuality.

We cannot rule out the influence of cultural differences in the relationship between personality and the use of dating apps. Young Spaniards may conceive of intimate relationships through this type of application differently from young people from other contexts where this phenomenon has been studied (Belgium [15]; United Kingdom, United States, and Canada [7]). In the absence of references in a young population similar to the Spanish one, we would need more studies, preferably cross-cultural, to test this hypothesis. Finally, dating app users may not actually differ from non-users in these antagonistic personality traits. If future research corroborated these results, we would have to banish the negative stereotype that is still associated with dating apps and their users [40].

The study has several limitations that need to be taken into account when interpreting the results. The use of dating apps has been evaluated without delving into the variety of uses, from those who used it on a single afternoon as a joke among friends to those who used it for months looking for a romantic relationship. Our estimation of current use is not a punctual prevalence, but with a timeframe of three months. The sample was mostly female, aged between 18 and 26, and coming from a single university. For this reason, it is difficult to generalize the results to the global population of university students and to young people of these ages who do not study at a university. Further, by grouping all the participating members of sexual minorities, we have lost information about the peculiarities of the use of dating apps and the personality patterns of these people depending on whether they are gay/lesbian, bisexual, or of other orientations. As for the instruments used to evaluate the Big Five and the Dark Core, lower levels of reliability were found than those of the original instrument validation studies [20,32]. For Agreeableness, Cronbach’s alpha was only 0.68. Reductions in reliability may have led to reductions in the estimated effect sizes and loss of statistical power. For the BFI-2 Short Form, in the German version [41], an even smaller value of Cronbach’s alpha was reported for this dimension (α = 0.65), so, apparently, these potential problems in reliability cannot be attributed to undetected problems with our sample. Moreover, our study shares with previous studies based on self-selected samples and self-reported measures the limitation due to the response and recall bias. In this sense, it would be interesting to carry out longitudinal studies that would allow evaluating evolution in personality traits, and their influence on the use of dating apps.

Some readers may consider as a problem the inclusion of instruments that have not been validated in Spanish samples, as we did with the BFI-2 or the Dark Factor of Personality–16. We do not share this concern. We cannot take for granted that an instrument that has shown adequate psychometric properties for specific use with a specific sample will show the same results with other samples. For example, if a validation effort is done for the development or adaptation of a Big Five questionnaire with a sample of university students from the north of Spain in 2018, we cannot guarantee that this instrument will be valid for a sample of nurses from the south of Spain in 2020. We have changed occupation, location, and time and we do not know if any of those changes may be relevant. The essential point is if there are any theoretical or empirical reasons to expect that the validity will be compromised with the intended sample. We consider that this is not the case for our Big Five measure with our sample. As Costa and McCrae have noted, “the [Big Five] factors are found in different age, sex, race, and language groups” [42]. From the same source, “they may be somewhat differently expressed in different cultures”. There are strong reasons to not expect this in our case. First, other measures of the Big Five have shown adequate properties in Spanish samples [43,44]. Second, the previous version of the BFI has also shown that “the Spanish BFI may serve as an efficient, reliable, and factorially valid measure of the Big Five for research on Spanish-speaking individuals” [45]. Third, the BFI-2 has shown adequate psychometric properties in different adaptations: German [41] or Russian [46]. In the case of dark personality, other measures of this construct have been previously validated into Spanish without any problem [47]. In any case, all our data are fully available for further research about the psychometric properties of those instruments (https://osf.io/wjuh6/).

Despite the above limitations, the study is considered to make some relevant contributions. First, information on the prevalence of dating apps use among Spanish university students has been provided, and this is one of the first studies to evaluate this phenomenon in Spanish-speaking youth. Second, both previous and current use have been taken into account, a novel aspect with respect to previous research that only evaluated the most recent use. In addition, this has been done in an unbiased sample, in which no attempt was made to overrepresent dating app users, as was the case in previous studies [2,3,18]. Thirdly, a profile of the dating apps user has been developed based on individual differences, with special relevance of sociodemographic variables (gender, age, sexual orientation, and relationship status), as all of them allowed for predicting use. Fourth, the traditional personality traits of the Big Five and dark personality have been simultaneously evaluated. Fifth, it has been found that, among personality dimensions, only Open-Mindedness can help to explain current use, although the contribution is of low magnitude.

## 5. Conclusions

The findings of this study have allowed us to plot a profile of the dating apps user based on some sociodemographic and personality characteristics. Men, older individuals, without a stable relationship (for current use), and belonging to a sexual minority are more likely to have used and/or to use such applications to relate with others and establish romantic relationships. However, the Big Five personality traits are not associated with past or recent use of dating apps, except for recent use being related to higher Open-Mindedness. Having dark or socially aversive personality traits does not help to explain the use of dating apps. The profile of previous and current users is largely equivalent.

In any case, the results obtained have revealed that the predictive power of personality for past and recent use of dating apps is very small compared to other individual features. In this sense, perhaps it would be appropriate to explore in more detail the influence of cognitive, motivational, and affective elements, more linked to situational and sociocultural influences, and therefore more changeable and adaptable to the characteristics of the environment, rather than focusing on more stable elements (i.e., traits). Although the individual’s behavior will depend on the continuous interrelationship of both elements, those who are more easily modifiable will be key aspects of mental and sexual health promotion programs in young people (e.g., negative emotions, low perception of risk of certain behaviors, etc.).

Dating apps, due to their huge use among adolescents and early adults, could be taken into account in the design of mental health protocols, including sexual and reproductive health strategies. For instance, as we found, people from sexual minorities are frequent users of dating apps. Meta-analytic evidence reported elevated risks for different mental health problems for sexual minority individuals [48]. Although dating apps can be useful to express their sexual identity and initiate a romantic relationship, these apps have also some characteristics (e.g., over-exposure) that could increase even more the risk for several mental health problems in vulnerable users (e.g., youth with low self-esteem). However, the popularity of dating apps could be used to promote sexual health among diverse and at-risk populations.

## Figures and Tables

**Table 1:** Bivariate relations of the different variables and descriptive statistics.

	1	2	3	4	5	6	7	8	9	10	11
	**Pearson *r***										
1. Negative Emotionality											
2. Extraversion	**–0.28**										
3. Open-Mindedness	0.01	**0.23**									
4. Agreeableness	**–0.18**	**0.17**	**0.17**								
5. Conscientiousness	**–0.20**	**0.24**	0.02	**0.19**							
6. Dark Core	0.03	**–0.06**	**–0.15**	**–0.59**	**–0.11**						
7. Age	**–0.05**	0.00	0.03	0.02	0.02	–0.02					
	**Cohen’s *d***										
8. Men	**–0.38**	**–0.17**	–0.01	**–0.42**	**–0.31**	**0.59**	0.02	**Pearson *r***			
9. Single	0.04	–0.08	0.03	**–0.20**	**–0.10**	**0.13**	**–0.27**	**0.15**			
10. Sexual minority	**0.37**	**–0.15**	**0.38**	**–0.12**	**–0.24**	–0.06	**–0.11**	–0.03	0.04		
	**ANOVA *R***							**Cramer’s *V***			
11. Apps use group	**0.07**	0.00	**0.10**	**0.08**	**0.10**	**0.08**	**0.24**	**0.12**	**0.24**	**0.25**	
Mean	19.02	19.49	22.94	23.16	19.83	27.61	20.60	0.30	0.53	0.30	–––
Standard Deviation	4.91	4.54	4.31	3.84	4.75	6.74	2.09	0.46	0.50	0.46	–––

Age, measured in years. Men: dummy variable where *women* = 0 and *men* = 1. Single: dummy variable where *in a relationship* = 0 and *single* = 1. Sexual minority: dummy variable where *heterosexual* = 0 and *sexual minority* = 1. ––– = Mean and standard deviation for apps use groups are not reported as this variable was nominal with three levels. Bold values correspond to statistically significant associations (*p* < 0.05).

**Table 2:** Means and standard deviations (for numerical variables), proportions (for categorical variables), and significance testing according to dating app use.

	Nonusers	Previous Users	Current Users		
	**Mean (Standard Deviation)**			***F***	***p***
Negative Emotionality	18.81 (4.95)	19.52 (4.82)	19.61 (4.71)	4.07	0.017
Extraversion	19.49 (4.49)	19.47 (4.73)	19.48 (4.61)	0.00	0.998
Open-Mindedness	22.74 (4.29)	22.97 (4.52)	24.01 (4.00)	8.04	<0.001
Agreeableness	23.34 (3.80)	22.91 (3.83)	22.49 (3.95)	5.20	0.006
Conscientiousness	20.11 (4.77)	19.25 (4.72)	18.94 (4.56)	7.94	<0.001
Dark Core	27.39 (6.62)	27.59 (6.08)	28.93 (7.94)	4.84	0.008
Age	20.31 (1.98)	21.70 (2.16)	20.86 (2.11)	53.85	<0.001
	**Proportion**			**χ^2^**	***p***
Women	0.75	0.15	0.10	23.25	<0.001
Men	0.65	0.17	0.18		
In a relationship	0.79	0.17	0.04	100.51	<0.001
Single	0.65	0.15	0.20		
Heterosexual	0.79	0.13	0.08	108.64	<0.001
Sexual minority	0.55	0.23	0.23		

Nonusers: participants reported having never used dating apps. Previous users: participants reported having used dating apps, but not in the last three months. Current users: Participants reported having used dating apps in the last three months. Age, measured in years. Proportions by row.

**Table 3:** Multinomial logistic regression analyses of use of dating apps.

	Apps Previous Users					Apps Current Users				
	***b***	***SE***	***OR***	**95% CI**	***p***	***b***	***SE***	***OR***	**95% CI**	***p***
Intercept	**–9.44**	**0.76**	**0.00**	**[0.00, 0.00]**	**<0.001**	**–8.66**	**0.88**	**0.00**	**[0.00, 0.00]**	**<0.001**
Negative Emotionality	0.15	0.08	1.16	[0.99, 1.36]	0.058	0.13	0.09	1.13	[0.95, 1.35]	0.159
Extraversion	0.15	0.08	1.16	[0.99, 1.35]	0.059	0.15	0.09	1.16	[0.98, 1.38]	0.087
Open-Mindedness	–0.07	0.08	0.93	[0.80, 1.08]	0.340	**0.20**	**0.09**	**1.22**	**[1.02, 1.45]**	**0.026**
Agreeableness	–0.05	0.09	0.95	[0.79, 1.13]	0.555	0.00	0.10	1.00	[0.82, 1.22]	0.989
Conscientiousness	–0.13	0.08	0.88	[0.76, 1.02]	0.085	–0.16	0.09	0.86	[0.72, 1.01]	0.067
Dark Core	–0.02	0.09	0.98	[0.82, 1.17]	0.816	0.17	0.10	1.19	[0.99, 1.44]	0.071
Age	**0.35**	**0.03**	**1.42**	**[1.33, 1.52]**	**<0.001**	**0.24**	**0.04**	**1.27**	**[1.17, 1.37]**	**<0.001**
Men	**0.36**	**0.17**	**1.44**	**[1.04, 1.99]**	**0.029**	**0.53**	**0.18**	**1.71**	**[1.21, 2.41]**	**0.002**
Single	0.20	0.15	1.22	[0.91, 1.62]	0.177	**1.87**	**0.21**	**6.48**	**[4.31, 9.73]**	**<0.001**
Sexual minority	**1.12**	**0.16**	**3.08**	**[2.27, 4.17]**	**<0.001**	**1.41**	**0.17**	**4.11**	**[2.95, 5.75]**	**<0.001**

*SE* = standard error; *OR* = odds ratio; CI = odds ratio confidence interval. All personality variables were standarized. Age, measured in years. Men: dummy variable where *women* = 0 and *men* = 1. Single: dummy variable where *in a relationship* = 0 and *single* = 1. Sexual minority: dummy variable where *heterosexual* = 0 and *sexual minority* = 1. Bold values correspond to statistically significant coefficients (*p* < 0.05). Nonusers was the reference group.

## Appendix A

**Table A1:** Spanish version of the Dark Core Scale [20].

Please read each statement and decide how much you agree or disagree with that statement. Note that there are no “correct” or “incorrect” answers to the statements. Please answer every statement, even if you are not completely sure of your response. If not specified otherwise, the items refer to your behavior (towards others) in general.	Por favor, lee cada oración y decide en qué grado estás de acuerdo o en desacuerdo con la misma. Recuerda que no hay respuestas correctas o incorrectas. Por favor, responde a cada afirmación, aunque no estés completamente seguro/a de tu respuesta. Las preguntas se refieren a tu comportamiento en general con los demás, a menos que se especifique lo contrario.
It is hard for me to see someone suffering.	Me resulta duro ver sufrir a alguien.
Payback needs to be quick and nasty.	La venganza debe ser rápida y cruel.
All in all, it is better to be humble and honest than important and dishonest.	En general, es mejor ser humilde y honesto que ser importante y deshonesto.
My own pleasure is all that matters.	Mi propio placer es lo único que importa.
I cannot imagine how being mean to others could ever be exciting.	NO puedo imaginar cómo ser desagradable con los demás puede ser excitante.
People who get mistreated have usually done something to bring it on themselves.	Las personas que son maltratadas generalmente han hecho algo para provocarlo.
Hurting people would make me very uncomfortable.	Hacer daño a alguien me haría sentir muy incómodo.
It’s wise to keep track of information that you can use against people later.	Es inteligente guardar información que puedas utilizar más adelante contra otras personas.
I feel sorry if things I do upset people.	Me siento mal/triste si las cosas que hago molestan a la gente.
People who mess with me always regret it.	La gente que se mete conmigo siempre se arrepiente.
Why should I care about other people, when no one cares about me?	¿Por qué debería preocuparme por otras personas cuando nadie se preocupa por mí?
I would like to make some people suffer, even if it meant that I would go to hell with them.	Me gustaría hacer sufrir a algunas personas, aunque eso significara hundirme con ellas.
Most people deserve respect.	La mayoría de la gente merece respeto.
I make a point of trying not to hurt others in pursuit of my goals.	Procuro NO hacer daño a otras personas mientras persigo mis objetivos.
I would be willing to take a punch if it meant that someone I did not like would receive two punches.	Estaría dispuesto a recibir un puñetazo si eso significara que alguien que NO me gusta recibiera dos puñetazos.
I avoid humiliating others.	Evito humillar a otros.

1 = Totalmente en desacuerdo / 2 = En desacuerdo / 3 = Neutral/Ni de acuerdo ni en desacuerdo / 4 = De acuerdo / 5 = Totalmente de acuerdo.
